# Phage-Derived Protein Induces Increased Platelet Activation and Is Associated with Mortality in Patients with Invasive Pneumococcal Disease

**DOI:** 10.1128/mBio.01984-16

**Published:** 2017-01-17

**Authors:** Rahajeng N. Tunjungputri, Fredrick M. Mobegi, Amelieke J. Cremers, Christa E. van der Gaast-de Jongh, Gerben Ferwerda, Jacques F. Meis, Nel Roeleveld, Stephen D. Bentley, Alexander S. Pastura, Sacha A. F. T. van Hijum, Andre J. van der Ven, Quirijn de Mast, Aldert Zomer, Marien I. de Jonge

**Affiliations:** a Department of Internal Medicine, Radboud Institute for Molecular Life Sciences, Radboud University Medical Center, Nijmegen, The Netherlands; b Center for Tropical and Infectious Diseases (CENTRID), Faculty of Medicine, Diponegoro University, and Dr. Kariadi Hospital, Semarang, Indonesia; c Laboratory of Pediatric Infectious Diseases, Department of Pediatrics, Radboud Institute for Molecular Life Sciences, Radboud University Medical Center, Nijmegen, The Netherlands; d Department of Medical Microbiology and Infectious Diseases, Canisius-Wilhelmina Hospital, Nijmegen, The Netherlands; e Department of Medical Microbiology, Radboud University Medical Center, Nijmegen, The Netherlands; f Department for Health Evidence, Radboud Institute for Health Sciences, Radboud University Medical Center, Nijmegen, The Netherlands; g Department of Pediatrics, Radboudumc Amalia Children’s Hospital, Radboud University Medical Center, Nijmegen, The Netherlands; h Wellcome Trust Sanger Institute, Pathogen Genomics Group, Hinxton, Cambridgeshire, United Kingdom; i Center for Molecular and Biomolecular Informatics, Radboud Institute for Molecular Life Sciences, Radboud University Medical Center, Nijmegen, The Netherlands; j Department of Infectious Diseases and Immunology, Faculty of Veterinary Medicine, Utrecht University, Utrecht, The Netherlands; Albert Einstein College of Medicine

## Abstract

To improve our understanding about the severity of invasive pneumococcal disease (IPD), we investigated the association between the genotype of *Streptococcus pneumoniae* and disease outcomes for 349 bacteremic patients. A pneumococcal genome-wide association study (GWAS) demonstrated a strong correlation between 30-day mortality and the presence of the phage-derived gene *pblB*, encoding a platelet-binding protein whose effects on platelet activation were previously unknown. Platelets are increasingly recognized as key players of the innate immune system, and in sepsis, excessive platelet activation contributes to microvascular obstruction, tissue hypoperfusion, and finally multiorgan failure, leading to mortality. Our *in vitro* studies revealed that *pblB* expression was induced by fluoroquinolones but not by the beta-lactam antibiotic penicillin G. Subsequently, we determined *pblB* induction and platelet activation by incubating whole blood with the wild type or a *pblB* knockout mutant in the presence or absence of antibiotics commonly administered to our patient cohort. *pblB*-dependent enhancement of platelet activation, as measured by increased expression of the α-granule protein P-selectin, the binding of fibrinogen to the activated αIIbβ3 receptor, and the formation of platelet-monocyte complex occurred irrespective of antibiotic exposure. In conclusion, the presence of *pblB* on the pneumococcal chromosome potentially leads to increased mortality in patients with an invasive *S. pneumoniae* infection, which may be explained by enhanced platelet activation. This study highlights the clinical utility of a bacterial GWAS, followed by functional characterization, to identify bacterial factors involved in disease severity.

## IMPORTANCE

The exact mechanisms causing mortality in invasive pneumococcal disease (IPD) patients are not completely understood. We examined 349 patients with IPD and found in a bacterial genome-wide association study (GWAS) that the presence of the phage-derived gene *pblB* was associated with mortality in the first 30 days after hospitalization. Although *pblB* has been extensively studied in *Streptococcus mitis*, its consequence for the interaction between platelets and *Streptococcus pneumoniae* is largely unknown. Platelets are important in immunity and inflammation, and excessive platelet activation contributes to microvascular obstruction and multiorgan failure, leading to mortality. We therefore developed this study to assess whether the expression of *pblB* might increase the risk of death for IPD patients through its effect on enhanced platelet activation. This study also shows the value of integrating extensive bacterial genomics and clinical data in predicting and understanding pathogen virulence, which in turn will help to improve prognosis and therapy.

## INTRODUCTION

*Streptococcus pneumoniae* or the pneumococcus is a frequent colonizer of the nasopharynx. In a minority of carriers, infection progresses to pneumococcal disease, which leads to an estimated 1.6 million deaths annually (1, 2). The largest clinical burden of invasive pneumococcal disease (IPD) is seen in young children and older adults, who present mostly with sepsis and meningitis. Case mortality rates are estimated to range from 11 to 30% in adults (3–5), with treatment becoming complicated due to the worldwide emergence of multidrug resistance (6). Therefore, it is of utmost importance to fully understand the pathogenic mechanisms of pneumococcal disease in order to improve the treatment and prognosis of critically ill patients.

Recently, the utilization of whole-genome sequencing and analyses for predicting and understanding pathogen virulence was highlighted (7). In this study, we performed a genome-wide association study (GWAS) on 349 pneumococcal draft genomes of blood isolates from patients who were admitted with IPD to two Dutch hospitals. We identified a significant association between 30-day mortality and the presence of *pblB*, encoding a platelet binding protein that was also reported to function in adhesion (8). In a subsequent functional study, we investigated the induction of phage-derived *pblB* expression by fluoroquinolones in *S. pneumoniae*. Lastly, we simulated *in vivo* conditions using an *ex vivo* whole-blood assay demonstrating the importance of PblB in enhancing platelet activation.

Platelets are an important part of the innate immune system and can interact with and be activated by *S. pneumoniae*. In sepsis, platelet activation and platelet-leukocyte complex (PLC) formation contribute to microvascular obstruction, tissue hypoperfusion, and finally multiorgan failure (9). The role of this phage-derived gene in the clinical outcomes of patients and the severity of their IPD, as well as the consequences of platelet activation, warrant further study.

## RESULTS

### *pblB* is an independent determinant of 30-day mortality in IPD patients.

**FIG 1:**
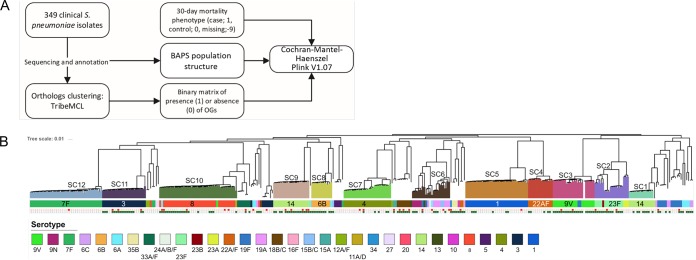
(A) Flow chart of the computational method used to identify the association between the presence of orthologous genes (OGs) and 30-day mortality; (B) phylogenic tree of the variable sites from the core genomes of all blood clinical isolates used in study. Phylogeny and sequence clustering were obtained from the work of Cremers et al. (37). Pneumococcal clades are colored according to their sequence clusters (SCs). Information on serotypes is indicated in the serotype legend. The filled red squares are those isolates that were derived from patients who died within 30 days of hospitalization. The *pblB* phage in our cohort was not present in serotype 7F and only barely in serotype 1, as indicated by the green squares. Filled squares, present; open squares, absent. Red indicates 30-day mortality. Dark green indicates the presence of *pblB*.

We conducted an unbiased association study for the presence or absence of pneumococcal genes and mortality within the first 30 days of hospitalization (Fig. 1A). Analysis was performed on 349 sequenced pneumococcal isolates collected from a clinical IPD cohort, which comprised strains from multiple lineages (10) (Fig. 1B). The GWAS was stratified for population structure, and the sequence cluster membership as determined by Bayesian analysis of population structure (BAPS) was used as a covariate in a Cochran-Mantel-Haenszel (CMH) test (11). The overall 30-day mortality within this IPD cohort was 11% (37/346; the outcome was unknown for 3 cases). We observed that of the 1,946 orthologous genes (OGs) of the pneumococcal accessory genome, *pblB* (OG_17) had a strong statistical correlation with 30-day mortality, with a Bonferroni-corrected *P* value of 0.00034, and was present in 48% of the 349 clinical isolates.

We identified *pblB* as the phage-derived gene potentially most relevant to the pathophysiology of IPD through its interaction with platelets, although it cooccurred with other phage genes (see Table S1 in the supplemental material). Sequence examination of a representative clinical isolate, PBCN0103, revealed that two copies of *pblB* were located within the same phage element next to OG_175 (holin) and OG_675 (hypothetical protein), both of which cooccurred with *pblB* and were also significantly associated with 30-day mortality (Fig. S1). In addition, OG_58, located in a phage operon different than those of the aforementioned genes, is also significantly associated with 30-day mortality (Table S1). Strikingly, these 4 OGs were present simultaneously in 168 out of 349 isolates (Fig. S2).

10.1128/mBio.01984-16.1 TABLE S1  Associations between patients’ mortality within the first 30 days of hospitalization (30-day mortality) and the presence and/or absence of genes in the pneumococcal isolates (represented as orthologous groups). The *P *values are Bonferroni adjustment corrected for multiple testing and stratified for population substructure using BAPS clusters (see Materials and Methods). Download TABLE S1, DOCX file, 0.1 MB. Copyright © 2017 Tunjungputri et al. 2017 Tunjungputri et al. This content is distributed under the terms of the Creative Commons Attribution 4.0 International license.

10.1128/mBio.01984-16.2 FIG S1  Part of the operon of a phage element. Genes are directed in reverse. Labels indicate gene products, and unlabeled arrows indicate hypothetical gene products. A total of 349 clinical isolates from patients with IPD were sequenced and annotated, and the genes were clustered into orthologous groups (OGs). Sequence examination of a representative clinical isolate, PBCN0103, revealed that two copies of *pblB* are located within a phage element next to OG_175 (holin) and OG_675 (hypothetical protein), both of which were also associated with 30-day mortality. Download FIG S1, DOCX file, 0.4 MB. Copyright © 2017 Tunjungputri et al. 2017 Tunjungputri et al. This content is distributed under the terms of the Creative Commons Attribution 4.0 International license.

10.1128/mBio.01984-16.3 FIG S2  Cooccurrence of *pblB* with other OGs associated with 30-day mortality. Thirteen OGs were statistically associated with 30-day mortality, of which four, namely, OG_17 (*pblB*), OG_175 (holin), OG_675 (hypothetical protein), and OG_58 (phage protein), were present simultaneously in 168 out of the 349 pneumococcal genomes. We tested whether these OGs cooccur within the same clinical isolates rather than being randomly distributed. The number of isolates that contain 0, 1, 2, 3 or all 4 of these OG(s) simultaneously was counted (“Observed”). The expected cooccurrence of the four OGs over the 349 genomes was mathematically calculated by multiplying the probability that a randomly picked genome contains 0, 1, 2, 3, or all 4 OGs with the total number of genomes (349) (“Expected”). Download FIG S2, DOCX file, 0.1 MB. Copyright © 2017 Tunjungputri et al. 2017 Tunjungputri et al. This content is distributed under the terms of the Creative Commons Attribution 4.0 International license.

Among the IPD cases caused by pneumococci containing the *pblB* gene (*pblB*^*+*^ strains), 27 out of 165 patients (16.4%) died within 30 days, compared to only 10 out of 181 patients (5.5%) infected with strains not containing the *pblB* gene (*pblB*-negative isolates) (*P* = 0.0011; odds ratio [OR], 3.3). In a subanalysis of cases who died without any limitations of medical treatment, 30-day mortality was 15/165 (9.1%) for those infected with a *pblB*^*+*^ strain and 6/171 (3.3%) for those infected with a *pblB*-negative strain, which remained statistically significant (*P* = 0.022; OR, 2.8). For all cases, the presence of *pblB* was an independent determinant of 30-day mortality (OR, 3.4; 95% confidence interval [CI], 1.5 to 7.6), besides a Charlson comorbidity index score (OR, 1.5; 95% CI, 1.2 to 1.7) and a finding of meningitis (OR, 4.6; 95% CI, 1.6 to 13.7). For pneumonia cases separately, in addition to the pneumonia severity index (PSI) score (OR, 1.4; 95% CI, 1.1 to 1.7) and the Charlson comorbidity score (OR, 1.02; 95% CI, 1.01 to 1.04), both designed to predict mortality, the presence of *pblB* was an independent risk factor for 30-day mortality (OR, 3.4; 95% CI, 1.2 to 9.5).

### Fluoroquinolones induced the expression of *pblB*.

**FIG 2:**
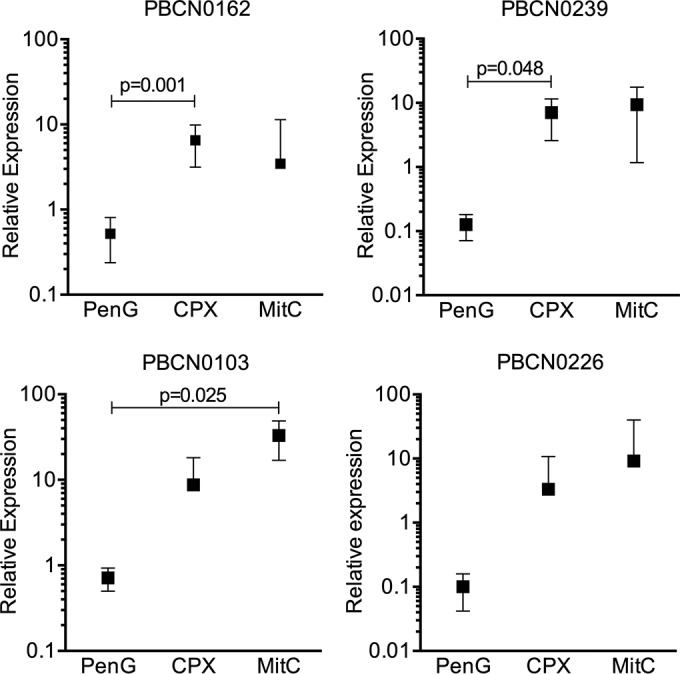
Sublethal doses of antibiotics induced pneumococcal expression of the *pblB* phage in culture medium. Induction of *pblB* expression after 2 h of incubation with sublethal doses of antibiotics was determined in 4 different pneumococcal clinical isolate strains (PBCN0162, PBCN0239, PBCN0103, and PBCN0226) by qRT-PCR by measuring levels of mRNA relative to the level of the control gene, *gyrA*. Data presented are means with 95% confidence intervals from three independent experiments.

It was unknown whether *pblB*-containing temperate pneumophages are specifically induced by this group of antibiotics *in vitro*. Therefore, different doses of ciprofloxacin (CPX) and levofloxacin (LVX), both belonging to the fluoroquinolone group of antibiotics, mitomycin C (MitC), and penicillin G (PenG; a beta-lactam antibiotic) were tested on three *pblB*-containing pneumococcal strains (PBCN0103, PBCN0226, PBCN0239) in Todd-Hewitt broth supplemented with yeast extract (THY) to determine the sublethal doses of the four antibiotics (data not shown). To confirm that the selected doses were not bactericidal, the number of CFU were determined after exposure to MitC, PenG, and the fluoroquinolones for 2 h at 37°C and 5% CO_2_ (Fig. S3). At the same time point, the difference in the levels of expression of *pblB* and *gyrA* was measured. The DNA cross-linking agent MitC was included as a positive control. Both the fluoroquinolones, CPX and LVX (data not shown), induced the expression of *pblB*, which appeared specific for this group of antibiotics, as the beta-lactam antibiotic PenG did not induce its expression. Furthermore, strong variation was found between the different pneumococcal strains (Fig. 2).

10.1128/mBio.01984-16.4 FIG S3  Average CFU values were determined after incubation of the four clinical pneumococcal strains (PBCN0162, PBCN0239, PBCN0103, PBCN0226) for 2 h at 37°C and 5% CO_2_ in THY medium with sublethal doses of the following antibiotics: mitomycin C (MitC), penicillin G (PenG), ciprofloxacin (CPX), and levofloxacin (LVX). The condition without antibiotics (−) was included as a negative control. Download FIG S3, DOCX file, 0.1 MB. Copyright © 2017 Tunjungputri et al. 2017 Tunjungputri et al. This content is distributed under the terms of the Creative Commons Attribution 4.0 International license.

### Simulation of the clinical conditions in a whole-blood *ex vivo* assay.

**FIG 3:**
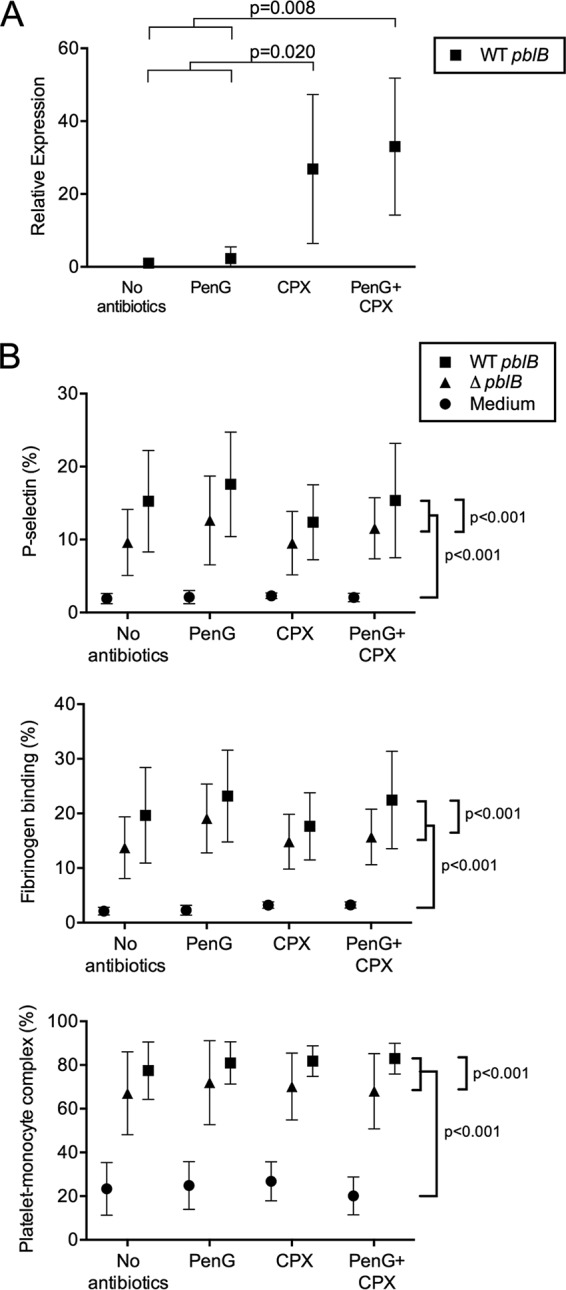
Live wild-type pneumococci in an *ex vivo* whole-blood assay showed increased expression of *pblB* upon exposure to fluoroquinolones and induced higher platelet activation than in the *pblB* knockout mutant, irrespective of antibiotic exposure. Live pneumococci (strain PBCN0162) containing a mutationally inactivated *pblB* gene (the Δ*pblB* mutant) or the wild type (WT *pblB*) were incubated in whole blood in the presence or absence of penicillin G (PenG), ciprofloxacin (CPX), or a combination of both. After 2 h of incubation at 37°C and 5% CO_2_, the expression of *pblB* was determined using qRT-PCR by measuring levels of mRNA relative to those in the control samples. One extreme outlier value, as determined by Grubbs’ test, in the CPX-exposed sample was excluded from panel A. Platelet expression of P-selectin, platelet-fibrinogen binding, and platelet-monocyte complex formation were measured in the same samples using flow cytometry and are expressed as percentages of positivity (B). Data presented are means with 95% confidence intervals from three independent experiments, with blood derived from a total of 6 human volunteers.

Of the 312 patients whose strains were sequenced and whose empirical treatment was known, 28% (*n* = 88) received only a beta-lactam, 4% (*n* = 11) received only fluoroquinolones, and 44% received a combination of a beta-lactam and a fluoroquinolone. To simulate the aforementioned clinical conditions, we incubated live pneumococcal strain PBCN0162, containing a mutationally inactivated *pblB* gene (Δ*pblB* mutant), or the wild type (WT) with and without antibiotics (PenG, CPX, and a combination of PenG and CFX) in whole blood, determined the expression of *pblB* using quantitative PCR (qPCR) (Fig. 3A), and measured in the same samples the activation of platelets. We were able to measure *pblB* expression of the WT pneumococci in the whole-blood samples without antibiotics (mean quantification cycle [*C*_*q*_] value, 30.6; 95% confidence interval, 29.5 to 31.7) and its increase in the presence of antibiotics. We first analyzed whether the different antibiotics significantly affected the WT- or Δ*pblB* bacterium-mediated platelet activation state in whole blood using a liner mixed model. We found that in all cases, stronger activation of platelets, together with higher platelet-monocyte complex (PMC) formation, was observed with WT pneumococci than with the Δ*pblB* mutant, which clearly indicates that PblB induces enhanced platelet activation irrespective of the exposure to antibiotics (Fig. 3B).

While PenG did not strongly induce the expression of *pblB* in THY medium-grown pneumococci (Fig. 2B), we observed PenG-dependent induction (~3-fold) of expression in whole blood (Fig. 3A). This might be caused by an indirect effect, a consequence of the bactericidal effect of PenG, leading to the production of reactive oxygen species (ROS), which have DNA-damaging effects, inducing the expression of *pblB*. Despite the fact that expression of *pblB* was much stronger in whole blood containing CPX, platelet activation was not increased accordingly, indicating close-to-maximum activation under these conditions.

## DISCUSSION

In the present study, a GWAS was performed using the sequences of 349 *S. pneumoniae* invasive-disease isolates to test for associations between the presence or absence of genes in the pneumococcal accessory genome and 30-day mortality. The presence of the phage-borne *pblB* gene was positively associated with 30-day mortality in patients with IPD. This finding suggested a role for *pblB* in pathogenesis and as the expected cause of death of patients with IPD. The presence of the *pblB* phage gene as a risk factor remained after adjustment for the local pneumococcal population structure using BAPS. We therefore speculate that similar studies in other areas with different pneumococcal populations would yield similar findings, although this requires confirmation by other studies. The *pblB* phage in our cohort was not present in strains of serotype 7F and only barely in strains of serotype 1, and these serotypes are associated with a lower risk of death than other serotypes (12).

Past observations reported that 75% of pneumococcal clinical isolates carry bacteriophages (pneumophages) (13), which may be distributed among pneumococcal isolates with different capsular serotypes, indicating that these mobile genetic elements are widely spread among clinically relevant pneumococcal strains (14). The hypothesis that bacteria acquire virulence properties from phages is widely accepted (15); however, there has been a paucity of data supporting the role of bacteriophages in the pathogenesis of *S. pneumoniae*-caused diseases. Interestingly, *pblB* cooccurred with two other genes in the phage element, one encoding a hypothetical protein and the other encoding holin, both of which were also found to be significantly associated with 30-day mortality. The simultaneous cooccurrence of these genes in almost 50% of our clinical isolates further indicates a functional link between PblB expression and 30-day mortality. The Holin protein is involved in the release of PblB and its mounting to the bacterial surface of *S. mitis* (16), allowing PblB’s interaction with cells and the propagation of platelet activation. Furthermore, PblB expression was also found to contribute to virulence in an *in vivo* rabbit model of infective endocarditis (17). These findings indicate that *pblB* has an important role in endovascular infection. In this cohort, of all *pblB*-positive strains, 2.4% have 3 open reading frames (ORFs) annotated as *pblB*, while 21.6% have 2 ORFs of various lengths annotated as *pblB*. In many genomes, the shorter *pblB* fragments are all located at the ends of contigs. It is likely either that these represent a single gene which has been fragmented due to an ~250-bp repeated sequence in the *pblB* gene or that the *pblB* gene is present in the genome in multiple copies, both resulting in contig breaks. As all these genes are annotated as *pblB* (OG_17), all were included in our association analysis.

Most patients in this cohort were treated with a combination of penicillin and ciprofloxacin, which represented a common first-line empirical antibiotic regimen for severe community-acquired pneumonia in The Netherlands (18). We therefore proceeded with *ex vivo* experiments in which live pneumococci were incubated in whole blood supplemented with penicillin or ciprofloxacin or a combination of the two to simulate clinical conditions. The wild-type pneumococci clearly demonstrated enhanced platelet activation. Interestingly, there were differences in platelet activation between knockout mutant and wild-type pneumococci even in the absence of high *pblB* induction by the antibiotics. This may be explained by a constitutive expression of *pblB*, which despite its low level was sufficient to induce platelet activation, as has been described for *S. mitis* (17).

*S. pneumoniae* has been shown to directly activate platelets, mainly through TLR2 (19), with FcγRIIA and integrin αIIbβ3 being involved in the amplification of bacterium-induced platelet activation (20). This leads to platelet degranulation and, subsequently, to the release of an array of chemokines and inflammatory mediators which may modulate not only their own function but also cells around them (21, 22). Our findings that whole-blood exposure to WT pneumococci results in higher platelet activation than does exposure to the *pblB* knockout mutant may explain why bacteremic patients infected with pneumococci containing the *pblB* gene have a higher chance of dying within 30 days. Approximately 20% increases from baseline values of platelet P-selectin expression and of PMC have been associated with adverse cardiovascular events and the acute phase of ischemic stroke (23, 24), and the increase in platelet activation associated with *pblB* in our *ex vivo* assays exceeded the aforementioned value. By causing enhanced platelet activation, bacteria can become engulfed in a septic thrombus and be protected from other cells of the immune system, allowing them to persist in the circulation (25). We speculate that *pblB*-enhanced platelet activation may confer this survival advantage to *S. pneumoniae*. On the other hand, the resulting excess of platelet activation together with platelet clumping, platelet-leukocyte and platelet-endothelium aggregation, and increased fibrin formation results in enhanced thrombo-inflammatory responses, microvascular obstruction, tissue hypoperfusion, and finally multiorgan failure in sepsis (26, 27). The increase in PMC formation predicts mortality in older septic patients (28), and platelet consumption associated with platelet activation in sepsis patients leads to thrombocytopenia, which has been shown to increase the risk of mortality (29–31). Autopsy was performed in only one case, which pointed at a myocardial infarction as the cause of death. In one other case, myocardial infarction was the most probable cause of death. In 16% of the cases, the cause of death was described as being due to respiratory failure or septic shock as a consequence of the primary diagnosis, and in the remaining cases, no cause of death was reported (32).

Our results have several potential clinical implications. First, we found that the presence of *pblB* was an independent determinant of 30-day mortality, which illustrates that a bacterial GWAS potentially identifies intraspecies variation related to clinical risks associated with human infection. Knowledge of the bacterial genotype might improve clinical management by increasing alertness for a particular disease manifestation, in this case, diffuse intravascular coagulation in *pblB*-positive IPD patients. However, as disease manifestations are generally the product of multiple covariates, the contributions of bacterial genotypes may vary across clinical settings. Second, our results demonstrated that fluoroquinolones induce high *pblB* expression. However, the presence of fluoroquinolones was not required by the *pblB*-expressing wild-type pneumococci to enhance platelet activation compared with that of the knockout mutant. Given that fluoroquinolones are frequently used in the management of community-acquired pneumonia for the coverage of atypical pathogens (33), sufficiently powered studies are needed to investigate the clinical outcomes of the interplay between the antibiotic regimen and *pblB* presence before drawing any conclusions. Third, our study further highlights the importance of platelet-bacterium interaction and platelet activation, both in providing a survival advantage for bacteria and in posing an increased risk of mortality in patients. There are more and more data on the use of platelet function inhibitors in sepsis; however, these results at times contradict one another (9). Platelet inhibition by the P2Y_12_ receptor antagonists reduces the release of proinflammatory mediators from the platelet α-granules (34). Taken together with our findings, the finding of a benefit of antiplatelet agents as adjunctive therapy in sepsis warrants further investigation.

The limitation of our study is the paucity of information on PblB protein expression on the pneumococcal surface. Previously, PblB of *S. mitis* was shown to function in adhesion by interacting with α2,8-linked sialic acid residues on platelet membrane gangliosides (35). More recently, Hsieh and colleagues showed that *pblB* knockout mutant pneumococci had decreased adherence to respiratory epithelial cells and platelets (8). Our study also adds to the research by showing that *pblB* may have additional effects, as we observed, on platelets in the bloodstream. Further work to demonstrate pneumococcal *pblB* expression at the protein level, as well as to identify its binding domain on platelets, is needed. The original objective of this study was to identify the association between the pneumococcal accessory genomes of the clinical isolates and the recorded clinical phenotypes of the patients. We cannot ascertain the cause of death for all patients who died, as it was not reported in the majority of cases and autopsy was performed in only one case. To the best of our knowledge, this is the only patient-based study which reveals the role of *pblB* gene expression in the pathogenesis of IPD based on an extensive analysis of both bacterial genomics and clinical data; it independently adds substantial evidence to only two previous studies on pneumococcal *pblB in vitro* and in mice (8, 36).

In conclusion, we have integrated genome sequencing and a GWAS with functional characterization to investigate the clinical role of *pblB*’s presence in the mortality of patients with IPD. A bacterial GWAS may be an important tool to study the potential predictive value of certain virulence genes. As genomic sequencing is increasingly being utilized, we believe that this integrated approach will assist greatly in elucidating the mechanisms of bacterial pathogenesis, leading to the development of novel diagnostics and new therapeutic approaches.

## MATERIALS AND METHODS

### Study population.

Consecutive patients hospitalized with a bacteremic pneumococcal infection at two Dutch hospitals between 2001 and 2011 were included in the study. Detailed clinical data were obtained on patient characteristics, clinical severity, treatment, and the course of disease. Corresponding blood culture isolates of *S. pneumoniae* were collected and serotyped as described before (10). For 349 of the isolated strains, sequencing, assembly of draft genomes, and annotation were done as previously described (37). This study was reviewed and approved by local medical ethical committees. All adult patients and healthy volunteers involved in this study provided written informed consent.

### Orthologous clustering and the GWAS.

Orthologous genes (OGs) from *S. pneumoniae* used in this study have previously been described by our group (37). Putative protein coding sequences were investigated using an “all-versus-all” protein BLAST (BLASTp), with a 10e−15 E value cutoff and a BLOSUM90 substitution matrix. The results were subsequently categorized into clusters of orthologous groups using TribeMCL (37, 38), resulting in a total of 3,021 OGs, 1,075 of which were conserved in all isolates in a single copy. The population (sub)structure (sequence clusters [SCs]) used for population stratification in the study have also been previously characterized (37). We based disease severity on mortality within the first 30 days of admission to the hospital and categorized the pneumococcal isolates into three groups: isolates derived from patients who died (*n* = 37), from patients who survived (*n* = 309), and from patients from whom the data were not captured (*n* = 3). The Cochran-Mantel-Haenszel (CMH) association statistics were employed to test the associations between the presence or absence of pneumococcal OGs and 30-day mortality, conditional on the bacterial population substructure as proposed by Bayesian analysis of population structure (BAPS; analysis 11). All associations were determined using PLINK (39). Candidate OGs were selected based on an association test with a *P* of <0.05 (with Bonferroni adjustment for multiple testing). Results were visualized using ITOL (40).

### Adjustment for covariates of mortality.

Potentially interesting covariates of 30-day mortality were analyzed using binary logistic regression analysis by likelihood ratio-based backward modeling; the pneumococcal OG and identified possible covariates were entered as explaining variables. Detailed statistical methods are described in Text S1 in the supplemental material.

10.1128/mBio.01984-16.5 TEXT S1  Adjustment for covariates of mortality. Download TEXT S1, DOCX file, 0.1 MB. Copyright © 2017 Tunjungputri et al. 2017 Tunjungputri et al. This content is distributed under the terms of the Creative Commons Attribution 4.0 International license.

### Induction of *pblB* expression by antibiotics.

Three isolates randomly selected from the group of deceased patients, containing the *pblB* gene, were selected: PBCN0103, PBCN0226, and PBCN0239. Different concentrations of mitomycin C, penicillin G, ciprofloxacin, and levofloxacin (all purchased from Sigma-Aldrich, Zwijndrecht, The Netherlands) were tested to determine the sublethal doses. The pneumococci were grown in THY medium to mid-log phase (optical density [OD], 0.3) and then diluted to an OD of 0.1, supplemented with 0.132 µg/ml mitomycin C, 0.0125 µg/ml penicillin G, 0.533 µg/ml ciprofloxacin, or 0.533 µg/ml levofloxacin, and grown for an additional 2 h at 37°C with 5% CO_2_. Subsequently, serial dilutions were incubated on blood agar plates (BD) and incubated overnight at 37°C with 5% CO_2_. Experiments were performed in triplicate to determine the expression of *pblB*. Mitomycin C was included as a positive control, as it was previously shown to induce *pblB* expression (41). After 2 h of growth, pneumococci were harvested by centrifugation. The supernatant was discarded, and a 2:1 volume of RNA Protect (Qiagen, Hilden, Germany) was added to the pellet. RNA was isolated using the RNeasy kit (Qiagen, Hilden, Germany) by following the manufacturer’s instructions. Residual DNA was removed with a DNase treatment using the Ambion Turbo DNA-free kit according to the manufacturer’s instructions (Ambion, Austin, TX). The qRT-PCR was performed as previously described by DeBardeleben et al. (41) using the following primers: HBgyrAF, AATGAACGGGAACCCTTGGT, HBgyrAR, CCATCCCAACCGCGATAC, pblB_F, TACAGCTGTGAAAGCCTTGG, and pblB_R, GATAGCCATCTGGATTCTCAGG.

### Construction of *S. pneumoniae* strain PBCN0162Δ*pblB*.

A directed gene deletion mutant of *S. pneumoniae* strain PBCN0162 was generated by allelic exchange of the target gene (*pblB*) with a spectinomycin resistance cassette (obtained from pR412T7), using the megaprimer PCR method; this resulted in PBCN0162Δ*pblB*. Briefly, flanking regions of ~500 bp, containing less than 150 bp of the coding sequence of the target genes, were amplified by PCR, with chromosomal DNA as the template. For each flanking region, the primer closest to the target gene (extension plus _L2 or _R2) contained an additional sequence complementary to primer PBpR412_L or PBpR412_R. In a second PCR, the PCR products of the two flanking regions and the antibiotic resistance cassette were combined, leading to incorporation of the antibiotic resistance cassette between the two flanking regions of the target gene, as previously described by Burghout et al. in 2007 (42). The primer sequences are provided in Table S2 in the supplemental material. Subsequently, the megaprimer PCR product was used for transformation of competent PBCN0162. Mutants, selected on blood agar plates containing spectinomycin, were assessed by colony PCR for recombination at the desired location on the chromosome. Chromosomal DNA was isolated from the mutants and used for transformation of competent strain PBCN0162. Gene inactivation was confirmed by quantitative real-time PCR gene expression analyses as described above (see “Induction of pblB expression by antibiotics”).

10.1128/mBio.01984-16.6 TABLE S2  List of primers. Download TABLE S2, DOCX file, 0.04 MB. Copyright © 2017 Tunjungputri et al. 2017 Tunjungputri et al. This content is distributed under the terms of the Creative Commons Attribution 4.0 International license.

### *Ex vivo* (whole-blood) assays.

Whole blood was obtained from healthy volunteers (*n* = 6) after informed consent using tubes anticoagulated with 3.2% citrate (BD Vacutainer, Becton, Dickinson, Plymouth, United Kingdom) and exposed to 1 × 10^7^ CFU/ml Δ*pblB* or WT pneumococci for 30 min at 37°C. Subsequently, either medium, PenG (0.0125 µg/ml), CPX (0.533 µg/ml), or a combination of PenG and CPX was added, and samples were incubated for 2 h at 37°C. RNA isolation and qRT-PCR were performed as described in the previous section. These whole-blood samples were also collected for measurement of platelet activation and platelet-monocyte complex (PMC) by flow cytometry.

### Measurement of platelet activation and PMC formation by flow cytometry.

Platelet activation was measured by whole-blood flow cytometry as previously described (43) by quantifying the platelet membrane expression of the α-granule protein P-selectin (CD62P) and the binding of fibrinogen to the activated αIIbβ3 receptor (GPIIbIIIa complex). The following antibodies were used to incubate samples from the whole-blood *ex vivo* assay: phycoerythrin (PE)-labeled anti-CD62P (Bio-Legend, San Diego, CA), fluorescein isothiocyanate (FITC)-labeled antifibrinogen (F0111-FITC; DAKO Ltd., High Wycombe, United Kingdom), and PC7-labeled anti-CD61 (platelet glycoprotein IIIa; Beckman Coulter, Inc., Miami, FL), the last as a platelet identification marker. The percentages of CD62P and fibrinogen in CD61-positive events were determined. Formation of PMC was measured by incubating samples with PC7-labeled anti-CD61 and PE-labeled anti-CD14 (a glycosylphosphatidylinositol [GPI]-linked membrane glycoprotein; Bio-Legend). After 20 min of incubation, OptiLyse B (Beckman Coulter, Inc., Fullerton, CA) was added to lyse erythrocytes. PMC formation was determined by quantifying the mean fluorescence intensity (MFI) of CD14^+^ cells that were also positive for the platelet identification marker CD61. All samples were measured using an FC500 flow cytometer (Beckman Coulter, Inc.).

### Statistical analyses.

Results from independent experiments (involving 6 donors) were pooled, and data are provided as means with 95% confidence intervals unless otherwise stated. A generalized linear mixed model with *post hoc* Bonferroni corrections was used to statistically analyze our experimental data. In the *in vitro* induction of *pblB* in culture medium, antibiotics were analyzed as a fixed effect on *pblB* expression, whereas interdonor variation was analyzed as a random effect (random intercept). For the whole-blood assay, the presence or absence of bacteria and the different antibiotics, as well as their interactions, were analyzed as fixed effects on platelet activation, and the interdonor variation was analyzed as a random effect (random intercept). All analyses were performed using SPSS version 20 (SPSS, Chicago, IL). The level of significance was set at a *P* of <0.05.
