# Supplementary material for: Phage-Derived Protein Induces Increased Platelet Activation and Is Associated with Mortality in Patients with Invasive Pneumococcal Disease
Source: mBio. 2017 Jan 17;8(1):e01984-16. doi: 10.1128/mBio.01984-16 (PMC5241397; doi:10.1128/mBio.01984-16)
Supplement: TABLE S1 [file mbo002173150st1.docx]

**Table S1.**

| **Associations between patients mortality within the first 30 days of hospitalization (30-day mortality), and the presence and/or absence of genes in the pneumococcal isolates (represented as orthologous groups;OG).** The *p-values* are Bonferroni corrected for multiple testing and stratified for population substructure using BAPS clusters [See material and methods]. | | | |
| --- | --- | --- | --- |
|  |  |  |  |
| **OG_ID** | **Annotation (Truncated)** | **Prevalence** | **Corrected p-value** |
| OG_675 | Hypothetical protein | 163 | 0.000228 |
| OG_17 | Phage hyaluronidase PblB | 166 | 0.000344 |
| OG_58 | Phage protein | 177 | 0.001017 |
| OG_1885 | Phage protein (prophage LambdaSa2) | 85 | 0.001923 |
| OG_2439 | Hypothetical protein | 41 | 0.00201 |
| OG_1220 | Phage protein | 93 | 0.002032 |
| OG_558 | ROK family protein | 318 | 0.002231 |
| OG_2259 | Hypothetical protein | 64 | 0.002756 |
| OG_866 | Phage protein | 106 | 0.003589 |
| OG_2298 | Phage protein | 27 | 0.003685 |
| OG_1029 | Hypothetical protein | 78 | 0.003737 |
| OG_175 | Phage holin | 171 | 0.004332 |
| OG_2232 | DNA-binding protein | 41 | 0.045225 |

| **Table S2. List of primers** | |
| --- | --- |
| **Target region** | **Primer** |
| CvdG_pblB_PBCN162_L1 | CGTTATCCAAATCGCAGGAC |
| CvdG_pblB_PBCN162_L2 | CCACTAGTTCTAGAGCGGCGGCTCTGTCAAATTGTCGTC |
| CvdG_pblB_PBCN162_R1 | AATTGGCGACGGCCATTTAC |
| CvdG_pblB_PBCN162_R2 | GCGTCAATTCGAGGGGTATCGACTATACCGCTTTAGTTCC |
| PBpR412_L | GCCGCTCTAGAACTAGTGG |
| PBpR412_R | GATACCCCTCGAATTGACGC |
