# Supplementary material for: Phage-Derived Protein Induces Increased Platelet Activation and Is Associated with Mortality in Patients with Invasive Pneumococcal Disease
Source: mBio. 2017 Jan 17;8(1):e01984-16. doi: 10.1128/mBio.01984-16 (PMC5241397; doi:10.1128/mBio.01984-16)
Supplement: FIG S1 [file mbo002173150sf1.docx]

**Supplemental Figure S1**


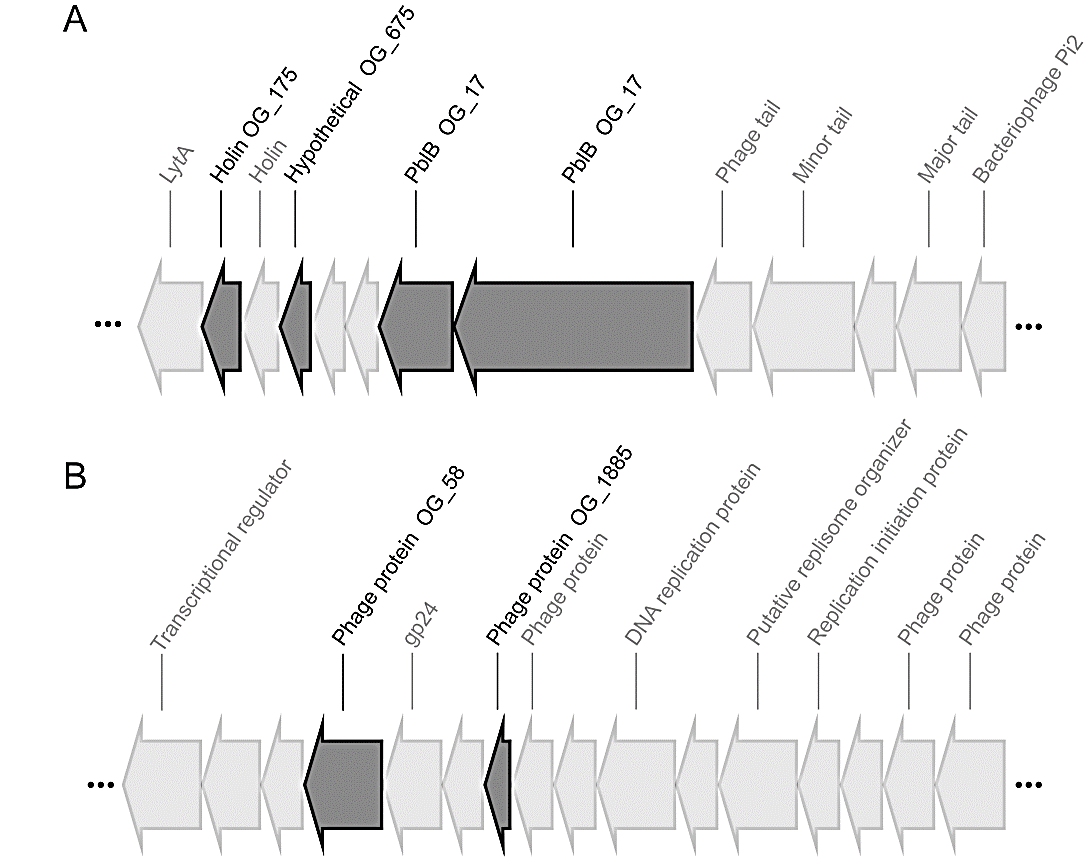


**Figure S1. Part of the operon of a phage element.** Genes are directed in reverse. Labels indicate gene products and unlabeled arrows indicate hypothetical gene products. A total of 349 clinical isolates from patients with IPD were sequenced, annotated and the genes were clustered into orthologous groups (OG). Sequence examination of a representative clinical isolate PBCN0103 revealed that two copies of *pblB* are located within a phage element in next to OG_175 (holin) and OG_675 (hypothetical protein), both of which were also associated with 30-day mortality.
