# Supplementary material for: Phage-Derived Protein Induces Increased Platelet Activation and Is Associated with Mortality in Patients with Invasive Pneumococcal Disease
Source: mBio. 2017 Jan 17;8(1):e01984-16. doi: 10.1128/mBio.01984-16 (PMC5241397; doi:10.1128/mBio.01984-16)
Supplement: FIG S2 [file mbo002173150sf2.docx]

**Supplemental Figure S2**

**Figure S2. Co-occurrence of *pblB* with other OGs associated with 30-day mortality.** Thirteen OGs were statistically associated with 30-day mortality, of which four, namely OG_17 (*pblB*), OG_175 (holin), OG_675 (hypothetical protein) and OG_58 (phage protein), were present simultaneously in 168 out of the 349 pneumococcal genomes. We tested whether these OGs co-occur within the same clinical isolates rather than being randomly distributed. The number of isolates that contain zero, one, two, three or all four of these OG(s) simultaneously was counted (“Observed”). The expected co-occurrence of the four OGs over the 349 genomes was mathematically calculated by multiplying the probability that a randomly picked genome contains 0, 1, 2, 3 or all 4 OGs with the total number of genomes (349) (“Expected”).
