# Supplementary material for: Phage-Derived Protein Induces Increased Platelet Activation and Is Associated with Mortality in Patients with Invasive Pneumococcal Disease
Source: mBio. 2017 Jan 17;8(1):e01984-16. doi: 10.1128/mBio.01984-16 (PMC5241397; doi:10.1128/mBio.01984-16)
Supplement: FIG S3 [file mbo002173150sf3.docx]

**Supplemental Figure S3**

**Figure S3.** Average CFU values were determined after incubation of the three clinical pneumococcal strains (PBCN0162, PBCN0239, PBCN0103, PBCN0226) for two hours at 37°C and 5% CO_2_ in THY medium with sub-lethal doses of antibiotics; mitomycin C (MitC), penicillin G (PenG), ciprofloxacin (CPX) and levofloxacin (LVX). The condition without antibiotics (-) was included as negative control.
