# Supplementary material for: Phage-Derived Protein Induces Increased Platelet Activation and Is Associated with Mortality in Patients with Invasive Pneumococcal Disease
Source: mBio. 2017 Jan 17;8(1):e01984-16. doi: 10.1128/mBio.01984-16 (PMC5241397; doi:10.1128/mBio.01984-16)
Supplement: TEXT S1 [file mbo002173150s1.docx]

**Method S1.**

**Adjustment for covariates of mortality.**

Certain patients had predetermined limitations of medical treatments, for example, opted not to be transferred to the intensive care unit. Therefore, the relation between OGs and 30-day mortality was also established separately for those who died after fully-applied treatment. For potential covariates of the associations between OGs and 30-day mortality, differences between patients with or without the OGs were assessed to decide which variables to include in the initial multivariable logistic regression model with 30-day mortality as dependent variable. The variables included were gender, age, year of inclusion, comorbidities (i.e. cancer, COPD, diabetes mellitus, liver-, renal-, cardiovascular- and cerebrovascular disease, Charlson comorbidity index score), clinical diagnosis, blood C-reactive protein level, presence of Systemic Inflammatory Response Syndrome (SIRS) and pleural effusion, Pneumonia Severity Index (PSI) score, admission to ICU, mechanical ventilation, and class of antibiotics administered. By manual stepwise backward elimination, the initial model was reduced to the final model, which only included covariates that contributed to the model with a p-value <0.10. These analyses were performed using IBM SPSS statistics version 23.
