# Supplementary material for: Phage-Derived Protein Induces Increased Platelet Activation and Is Associated with Mortality in Patients with Invasive Pneumococcal Disease
Source: mBio. 2017 Jan 17;8(1):e01984-16. doi: 10.1128/mBio.01984-16 (PMC5241397; doi:10.1128/mBio.01984-16)
Supplement: TABLE S2 [file mbo002173150st2.docx]

| **Table S2. List of primers** | |
| --- | --- |
| **Target region** | **Primer** |
| CvdG_pblB_PBCN162_L1 | CGTTATCCAAATCGCAGGAC |
| CvdG_pblB_PBCN162_L2 | CCACTAGTTCTAGAGCGGCGGCTCTGTCAAATTGTCGTC |
| CvdG_pblB_PBCN162_R1 | AATTGGCGACGGCCATTTAC |
| CvdG_pblB_PBCN162_R2 | GCGTCAATTCGAGGGGTATCGACTATACCGCTTTAGTTCC |
| PBpR412_L | GCCGCTCTAGAACTAGTGG |
| PBpR412_R | GATACCCCTCGAATTGACGC |
